# 3D electroanatomical mapping is less sensitive to atrial remodeling in estimation of true left atrial volume than echocardiography

**DOI:** 10.1186/s12880-018-0276-2

**Published:** 2018-09-18

**Authors:** Zdenka Fingrova, Josef Marek, Stepan Havranek, Lukas Lambert, Petr Kuchynka, Ales Linhart

**Affiliations:** 1 2nd Department of Medicine – Department of Cardiovascular Medicine, First Faculty of Medicine, Charles University and General University Hospital, U Nemocnice 2, 128 08 Prague, Czech Republic; 2 Department of Radiology, First Faculty of Medicine, Charles University and General University Hospital, U Nemocnice 2, Prague, 128 08 Czech Republic

**Keywords:** Atrial fibrillation, Echocardiography, Cardiac CT, Left atrial volume, Electroanatomic mapping, Catheter ablation

## Abstract

**Background:**

Left atrial (LA) enlargement has been identified as a predictor of worse clinical outcome after catheter ablation for atrial fibrillation (AF). We investigated the correspondence of LA size parameters assessed by echocardiography, CT and 3D electroanatomical mapping in patients with AF treated by catheter ablation.

**Methods:**

We analyzed echocardiographic LA volume measurements by disc summation method (LAV_DISC_), computed tomography (LAV_CT_) and 3D electroanatomical mapping (LAV_CARTO_) in 100 pts. (71% males; aged 63 ± 8 years; paroxysmal AF in 55% of patients).

**Results:**

Mean LAV_DISC_ was 83 ± 25 ml (median: 115; IQR: 98–140 ml), mean LAV_CT_ was 120 ± 34 ml (median: 115; IQR: 98–140 ml) and mean LAV_CARTO_ was 123 ± 36 ml (median: 118; IQR: 99–132 ml). Pearson’s correlation coefficient between LAV_DISC_ a LAV_CT_ was 0.6 (*p* < 0.0001) and between LAV_CARTO_ and LAV_CT_ was 0.79 (p < 0.0001). There was a significant difference between the two correlation coefficients (*p* < 0.004). The absolute difference between LAV_CARTO_ and LAV_CT_ (3.5 (95% CI -42 – 43) ml) was significantly lower (*p* < 0.0001) as compared to LAV_DISC_ and LAV_CT_ (− 39 (95% CI -102 – 24) ml). In opposite to LAV_DISC_, the bias between LAV obtained by CT and CARTO did not differentiate according to presence of spherical remodeling (1.7 ± 28 vs. vs. 5.1 ± 31 ml). Only presence of sinus rhythm was significant and independent covariate of the difference between CARTO and CT-derived LAVs by multivariate regression analysis.

**Conclusions:**

Even though LA volumes evaluated by 3D-electroanatomical mapping have quite good accuracy, the precision is low. For volumes estimated by echocardiography, both precision and accuracy are low.

## Background

Catheter ablation has been established as an effective therapy in selected patients with atrial fibrillation (AF) [1, 2]. Electroanatomical mapping and 3D reconstruction of the left atrium (LA) has been introduced as routine method for navigation during catheter ablation for AF. Evaluation of LA size, which has been detected as a predictor of catheter ablation outcome [3, 4], is substantial.

In everyday practice echocardiography is the most dominant method in estimation of LA size. However its predictive value in estimation of real LA volume (LAV) is not optimal [5–10]. Different methods for the assessment of LAV by use of 2D-echocardiography (ECHO) (e.g. area-length, prolate-ellipsoid method or disc method in single or biplane variant) have been introduced [5–7, 11, 12]. Despite these methods are more accurate in estimation of true LA size than isolated LA diameter [5–7, 11, 12], they still systematically underestimate volumes obtained by 3D-ECHO, CT or MRI [5–10]. Two more studies have shown the substantial disagreement between 2D-ECHO-based LAV and LAV obtained by 3D electroanatomic mapping and clinical covariates influencing this discrepancies in patients with non-valvular AF [13, 14].

In contrast to 2D-ECHO indices, some reports have demonstrated high degree of agreement between LA electroanatomical map and CT-assessed LA anatomy [15–17] or 3D echocardiography [18].

There are only limited data about factors that influence the relationship between LAV obtained by electroanatomical mapping and CT. Moreover, previous studies demonstrating correspondence of morphology of LA assessed by 3D electroanatomical mapping and CT angiography, have not compared LAV directly [15–17]. We hypothesized that LAV obtained by electroanatomic 3D reconstruction would assess LAV in better agreement with CT than 2D-echocardiography. We also hypothesized that some clinical or morphological characteristics of LA influencing this variance could be detected. We investigated this hypothesis in real-world population of patients with non-valvular AF scheduled for catheter ablation in whom electroanatomic 3D reconstruction and CT angiography of the LA was performed.

## Methods

Consecutive patients, who were scheduled for first catheter ablation for AF with CT registration and had complete echocardiographical evaluation of LA size at one cardiology center between May 2013 and December 2015, were included to retrospective analysis. The data were collected in a dedicated registry. The study was approved by the local ethic committee. All patients gave their written informed consent to participate in the study.

### Computed tomography

Computed tomography (CT) was performed in supine position at end-inspiration on a 256-slice CT scanner (Brilliance iCT, Philips Healthcare, Best, The Netherlands) as a prospective ECG-triggered axial (step & shoot) end-diastolic (78% of the R-R interval) acquisition. The scan was initiated by bolus tracking with a 180HU threshold in a region of interest placed in LA during injection of 65 ml of iodinated contrast material (Iomeron 350, Bracco Imaging Deutschland, Germany) at a flow rate of 4.0 ml/s followed by 60 ml saline chaser at 4.0 ml/s. The acquisition parameters were: tube voltage, 120 kV; tube current, 708 mA; collimation, 128 × 0.625 mm; rotation time, 0.27 s. The images were reconstructed in 0.9 mm thin sections with 50% overlap using reconstruction algorithm XCA and iterative reconstruction technique (iDOSE4, level 4). Raw data were processed by use of dedicated Philips Intelli Space portal. CT LA_long_ and CT LA_short_ were LA diameters measured in apical four chamber projection. LA was then centered on all three cutting planes and CT LA_cranio-caudal_, CT LA_antero-posterior_ and CT LA_transversal_ diameters were measured. The diameters were obtained as the largest diameter in given projection. LAV_CT_ was derived from segmented LA cavity without inclusion of left atrial appendage. Index of sphericity (IS) was calculated with following equation: IS = (1 - coefficient of variation of sphere) * 100. Coefficient of variation of sphere was defined as average radius standard deviation divided by average radius. Average radius was calculated as mean of radius in three dimensions: antero-posterior, cranio-caudal and transversal [19]. IS was used as marker of atrial remodeling, when data were dichotomized according to median of IS.

### 3D Electroanatomical mapping

3D electroanatomical mapping of LA was completed in standardized manner at the beginning of whole ablation procedure. We have applied protocol described in previous study [13]. A 3D electroanatomic mapping system (CARTO 3, Biosense-Webster Inc., Diamond Bar, CA, USA) and manual catheter navigation was used for 3D reconstruction of the LA cavity. Uniformly distributed mapping points at end-diastolic phase (time interval from -150 ms to -10 ms before QRS complex) were acquired at sites with stable endocardial contact. No mapping points behind pulmonary vein ostia were included to the map. Attention was paid to precise delineation of the mitral annulus. The orifice and proximal part of LA appendage was always mapped. Intracardiac echocardiography was used to identify and mark the critical structures. A 3D virtual map of the LA was constructed by software interpolations over the co-ordinates of multiple endocardial tags. LAV_CARTO_ was calculated using a built-in computational function of the Biosense software prior to merging CARTO and CT reconstruction of atrium.

### Echocardiographic examination

Transthoracic echocardiographic examinations were indicated prior to the catheter ablation according to the guidelines of American Society of Echocardiography 2005 valid at the time of initiation of data collection [12, 20]. The protocol was analogous to previously published methodology [13]. The LA diameter (LAD) was obtained in M-mode and defined as antero-posterior, end-systolic linear dimension in the parasternal long-axis projection using 2D guidance. The LAV_ELLIPSOID_ was calculated by the prolate-ellipsoid method, which used three LA orthogonal diameters in ventricular end-systole / atrial diastole (LAD and two orthogonal diameters in the apical 4-chamber view). A standardized biplane disc method (in apical 4-chamber and apical 2-chamber view) was applied to assess LAV_DISC_. The tracing of LA cavity was performed without including of pulmonary vein and left atrial appendage.

### Statistical analysis

Continuous variables were expressed as means with standard deviations after testing for normality (Shapiro-Wilk’s test) and compared by the 2-tailed t-test for independent samples. Not normally distributed variables or ordinary data were expressed as medians, interquartile ranges and compared by two-tailed Mann-Whitney *U* test. Categorical variables were expressed as percentages and compared by χ2–test. Pearson’s correlation, Bland-Altman analysis and multivariate linear regression (for all univariately different variables with *p* ≤ 0.2) were used to analyze the relationship between 2D-ECHO-based LAV indices or LAV_CARTO_ (together with other clinical covariates) as independent variables and LAV_CT_ as dependent variable. The difference between correlation coefficients was computed using the r-to-Fisher-z transformation. A *p*-value < 0.05 was considered significant. All analyses were performed using the STATISTICA vers.12 software (Statsoft, Inc., Tulsa, USA).

## Results

**Fig. 1:**
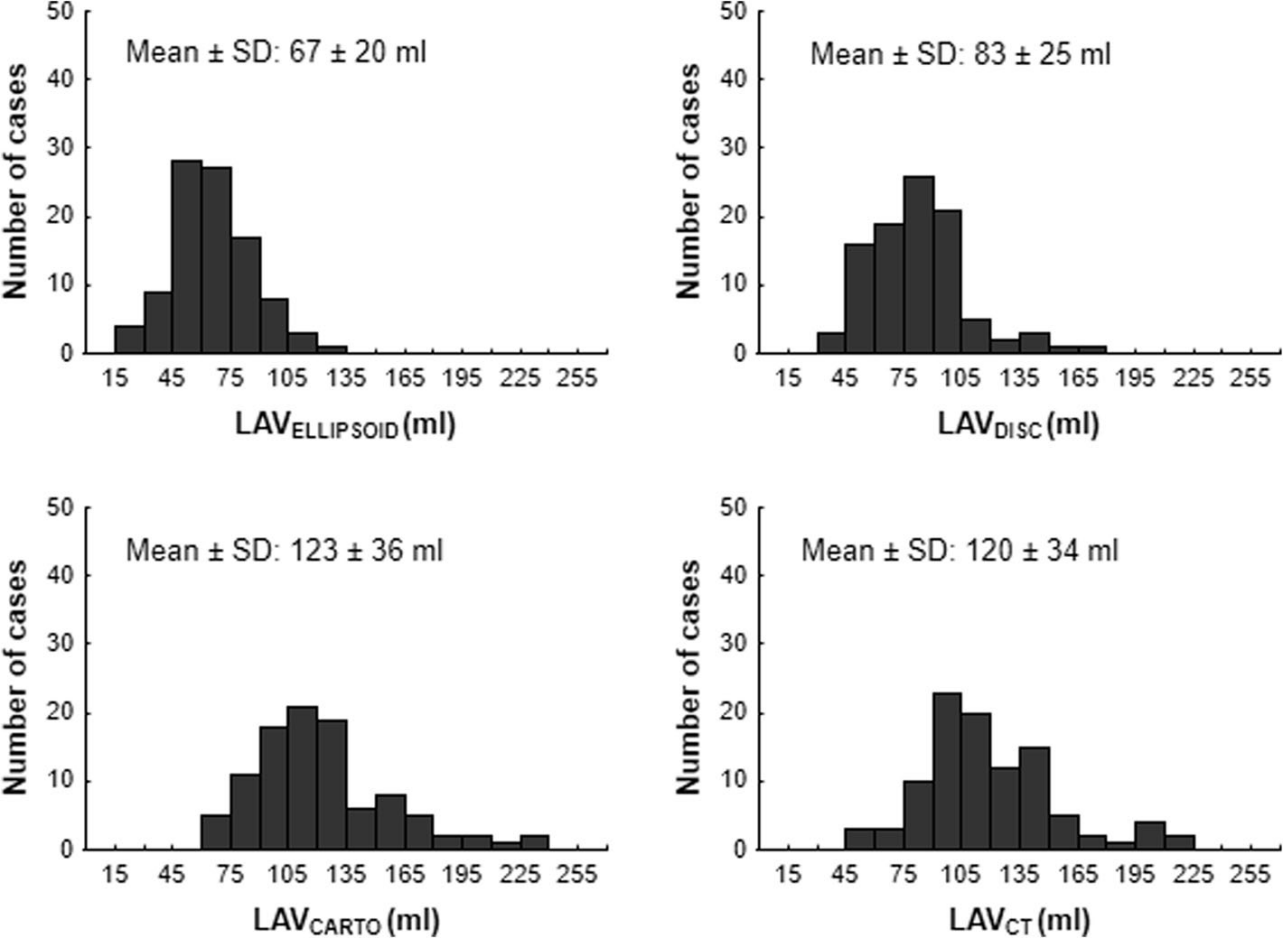
Distribution of left atrial volumes. Abbreviations: SD – standard deviation; IQR – interquartile range; LAV_ELLIPSOID_ – left atrial volume (LAV) assessed by the prolate-ellipsoid method; LAV_DISC_ – LAV assessed by the biplane disc method; LAV_CARTO_ – CARTO-derived LAV; LAV_CT_ – LAV assessed by computed tomography

One hundred thirty-six patients ablated for AF and had undergone CT prior to intervention were screened to the study. 36 patients were excluded for insufficient echocardiographical data (missing or low quality of recordings). A total 100 patients (aged 63 ± 8 years; 71% males) were analyzed. Paroxysmal AF was manifested in 55 (55%) patients. Rest of subjects (45%) had persistent or long-standing persistent AF, respectively. Baseline characteristics of the total population are shown in Table 1. Females were significantly older than males 66 ± 8 vs. (62 ± 8 years, *p* = 0.03). Out of all AF classified as non-paroxysmal, only 38 (84%) were in AF. All patients with AF during CT scan had AF also when ECHO or electroanatomical mapping were obtained. AF initiated during electroanatomical mapping in 3 (5%) subjects with SR during CT and ECHO. The distributions of LAV indices are illustrated in Fig. 1.

**Table 1:** Baseline characteristics

*N* = 100	Mean ± SD or n (%)	Median	IQR
Age (years)	63 ± 8	–	–
Males	71 (71%)	–	–
Paroxysmal AF	55 (55%)	–	–
Present sinus rhythm	62 (62%)	–	–
Hypertension	71 (71%)	–	–
Diabetes mellitus	13 (13%)	–	–
Structural heart disease	23 (23%)	–	–
Congenital heart disease	0 (0%)		
CHADS_2_ score	–	1	1; 2
CHA_2_DS_2_-VASc score	–	2	1; 3
LV EF (%)	–	62	56; 66
LAVi (ml/m^2^)	40 ± 11	–	–
CARTO mapping points	135 ± 66	–	–
ECHO LAD (PLAX) (mm)	46 ± 6	–	–
ECHO LA_long_ (A4C) (mm)	58 ± 8	–	–
ECHO LA_short_ (A4C) (mm)	47 ± 7	–	–
CT LA_long_ (A4C) (mm)	52 ± 8	–	–
CT LA_short_ (A4C) (mm)	43 ± 6	–	–
CT LA_Cranio-caudal_ (mm)	61 ± 7	–	–
CT LA_Antero-posterior_ (mm)	52 ± 7	–	–
CT LA_Transversal_ (mm)	69 ± 7	–	–
LA Sphericity index	81 ± 7	–	–

*AF* atrial fibrillation; *LV EF* left ventricular ejection fraction; *LAVi* left atrial volume indexed to body surface area; *CT* computed tomography; *LAD* left atrial diameter in parasternal long-axis view (PLAX); *LA* – left atrium; A4C – Apical four chamber view. *SD* standard deviation; *IQR* interquartile range

**Fig. 2:**
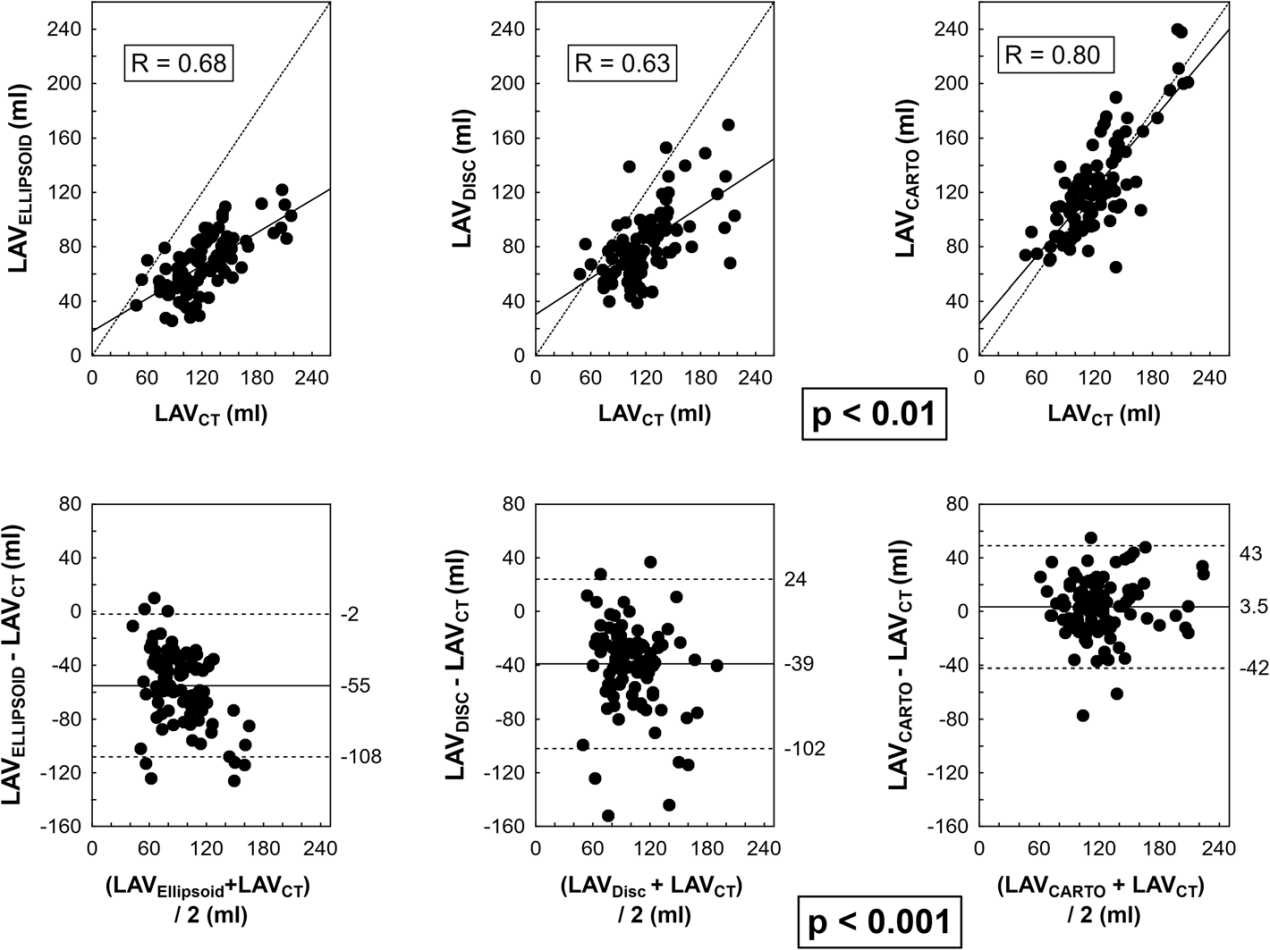
Correlation and agreement between 2D-ECHO-based, CARTO-derived and CT-derived left atrial volumes. Caption: Upper row: Pearson’s correlation. Lower row: scatterplots for absolute differences between 2D-ECHO-based or CARTO-derived LAV versus CT-derived LAV. Abbreviations as in Fig. 1. Upper raw: Dotted line – identity line; full line – regression line. Bottom raw: Full line – bias; dashed line – limits of agreement (±1.96 standard deviation). Significance of difference in relationship of 2D-ECHO-based and CARTO-derived to CT-derived volumes is highlighted

**Fig. 3:**
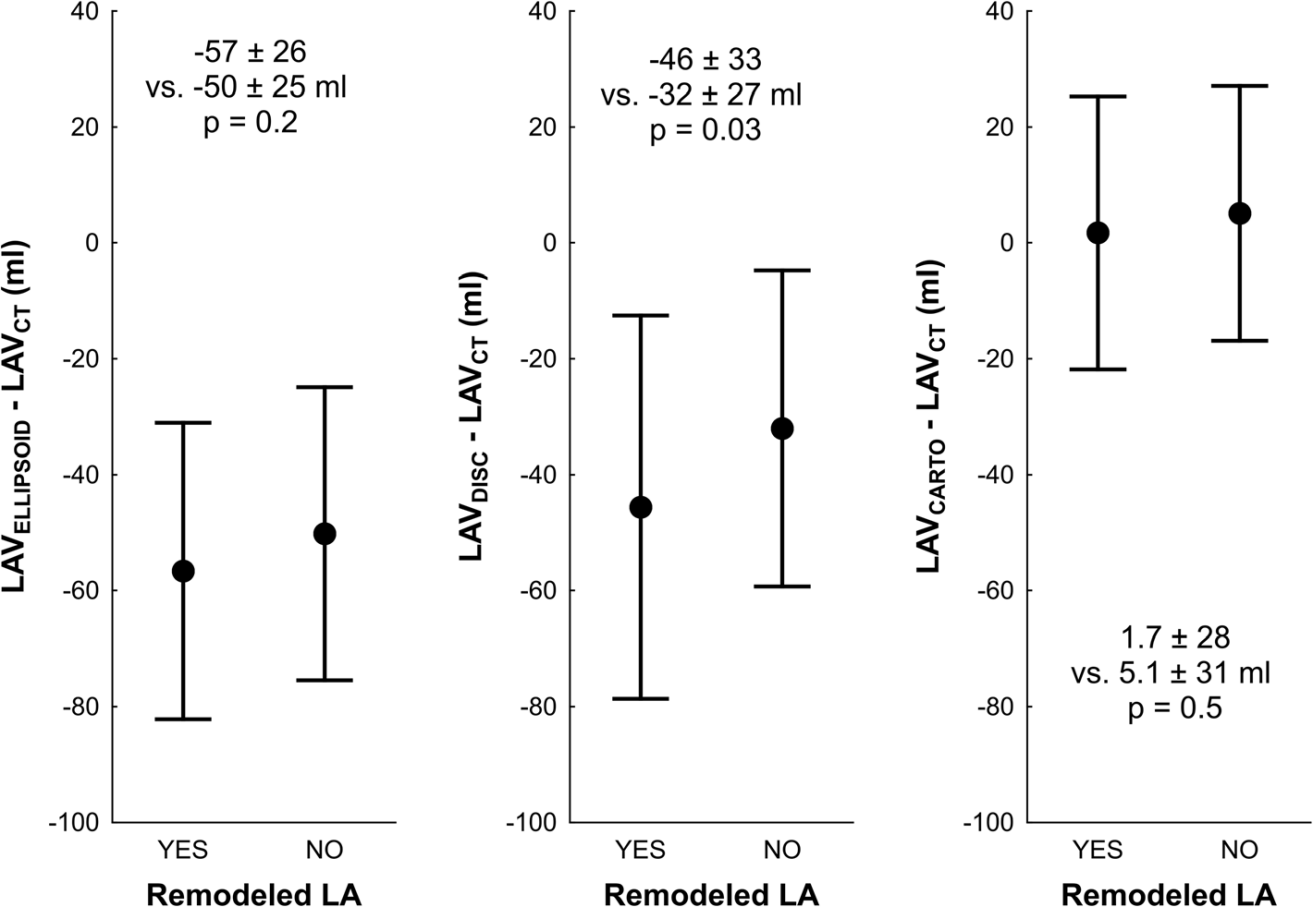
Differences between 2D-ECHO-based, CARTO-derived and CT-derived left atrial volumes according to remodeling of left atrium. Caption: Left atrium remodeling was defined according to sphericity index obtained from CT. Atrias were dichotomized according to median of sphericity index. The points and whiskers represent mean and ± standard deviation. Abbreviations as in Fig. 1

The results of simple regression between ECHO-based LAV parameters, CARTO based LAV and LAV_CT_ are shown in Fig. 2. There was a more positive correlation between LAV_CARTO_ and LAV_CT_ compared to LAV_DISC_ LAV_CT._ (*p* < 0.05). The correlation coefficient of LAV_ELLIPSOID_ and LAV_CT_ did not differ from coefficient between LAV_CARTO_ and LAV_CT_ or LAV_DISC_ and LAV_CT._ LAV_ELLIPSOID_ and LAV_DISC_ underestimated LAV_CT_ with an absolute bias (± 1.96 standard deviation) of − 55 (− 108; − 2) ml and − 39 (− 102; + 24) ml, respectively. LAV_CARTO_ was even more comparable to LAV_CT_ with an absolute bias 3.5 (− 42; + 43) ml; *p* < 0.001 for difference between indices based on 2D-ECHO LAV and based on LAV_CARTO_.As depicted in Fig. 3, the bias between LAV_DISC_ and LAV_CT_ differ according to presence of spherical remodeling of LA (defined as IS above / below median). This sensitivity to atrial remodeling was not recognized in LAV_ELLIPSOID_ or LAV_CARTO_.

**Fig. 4:**
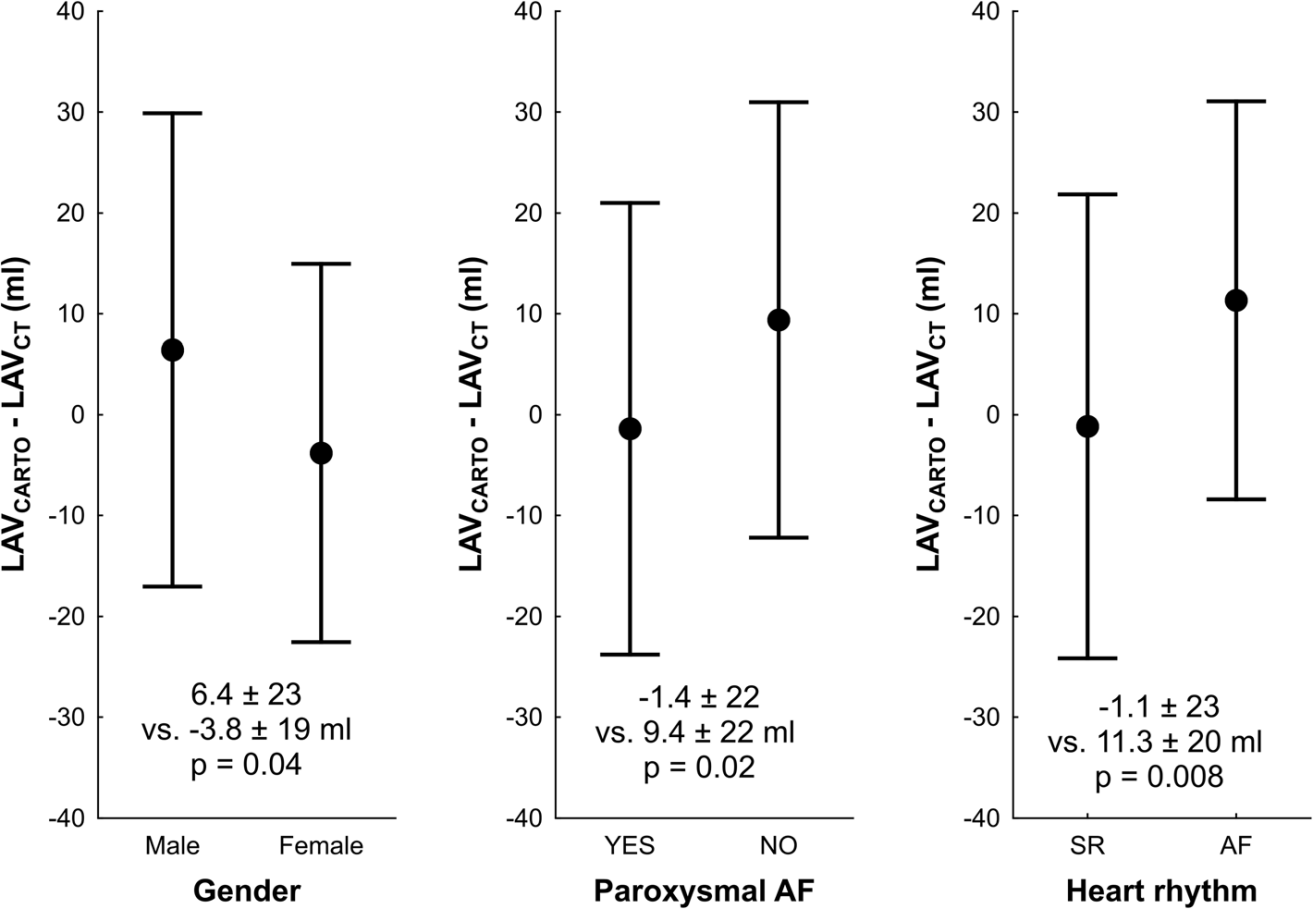
Differences between CARTO-derived and CT-derived left atrial volumes according to clinical covariates. Caption: The points and whiskers represent mean and ± standard deviation. Abbreviations as in Fig. 1

The differences between LAV derived from CARTO and CT were significantly larger in males, patients without paroxysmal AF and subjects without SR during CT scan in univariate manner (Table 2 and Fig. 4). Other tested clinical parameters (age > 65 years, arterial hypertension, diabetes, presence of structural heart disease) were not statistically significant. Only presence of SR was significant and independent covariate of the difference between CARTO and CT-derived LAVs by multivariate regression analysis, when all significant and near to significant parameters (*p* ≤ 0.2) in univariate analysis were included (Table 2).

**Table 2:** Univariate and stepwise forward multivariate regression analysis of determinants of discrepancy between left atrial volume obtained by computed tomography and 3D electroanatomical mapping

	Univariate analysis			Multivariate analysis	
	YES (ml)	NO (ml)	*p* value	Regression coefficient (95% CI)	*p* value
Intercept	–	–	–	7 (−3; 18)	0.16
Male gender	6.4 ± 23	−3.8 ± 19	0.04	5 (− 1; 18)	0.08
Paroxysmal AF	−1.4 ± 22	9.4 ± 22	0.02	−5 (− 16; 6)	0.34
Age > 65 years	− 0.8 ± 22	6.0 ± 23	0.15	−0.1 (− 0.7; 0.4)	0.67
Arterial hypertension	1.6 ± 23	8.1 ± 21	0.20	−5 (− 15; 6)	0.36
Diabetes mellitus	−3.5 ± 22	4.9 ± 21	0.18	8 (−6; 21)	0.27
Structural heart disease	−3.6 ± 28	5.7 ± 20	0.09	−9 (−20; 2)	0.09
SR during CT	−1.1 ± 23	11.3 ± 20	0.008	−12 (−21; − 3)	0.008

Values are in format mean ± SD. In multivariate analysis YES = 1; NO = 0. *LAV*_*CARTO*_ left atrial volume obtained by electroanatomical mapping; *SR* sinus rhythm; *CI* confidence interval

## Discussion

The analysis realized in a real-world population of patients with non-valvular AF verified better agreement between CARTO derived LAV and reference LAV assessed by CT than between 2D-ECHO-based LAV indices and the same reference. The major outcome of the study is that, in contrast to 2D-ECHO-based LAV obtained by disc biplane method, the bias between LAV_CARTO_ and LAV_CT_ was less sensitive for spherical remodeling. However, despite smaller bias between LAV obtained by CARTO and CT, LA size can be still over- or underestimated by the CARTO. Presence of SR during CT scan was identified as independent covariate in prediction of difference between CARTO and CT derived LAV.

The discrepancy between LA size obtained by various methods has been observed previously. As CT, MRI or 3D-ECHO rectified insight of the LA anatomy as an asymmetrical 3D structure [7–10], the sphericity simplification of echocardiographic methods results in underestimation of the LAV by 2D-ECHO [5, 6, 9, 12], especially in enlarged atria [6, 13]. Atrial remodeling is associated with a change of LA shape, which caused the standard geometric models even more inaccurate. Such variances include, for example, a trapezoidal LA shape [21], dilatation of the pulmonary vein antrum area [22], LA roof re-shaping [23], enlargement of the anterior portion of the LA [24], and spherical remodeling of the LA [19]. Even the well-validated biplane 2D-based methods, including biplane LAV_DISC_ method, systematically underestimated LAV when compared with 3D-ECHO, MRI, CT or CARTO [9, 10, 13, 14].

Even though biplane disc method was allowed in estimation of LAV by previous guidelines [12], the correspondence of LAV_DISC_ to LAV_CT_ had been low and still dependent on spherical remodeling demonstrated by simplified IS. It responds to expectation that the missing of constant proportions between the orthogonal axes in remodeled and spherically deformed LA more likely affect LA size parameters based on geometrical models (including 2D-ECHO derived methods) instead of direct measurement.

In contrast to disc biplane method, bias between LAV_ELLIPSOID_ and LAV_CT_ was not changed significantly when LA atrium was spherically remodeled. This phenomenon stays in parallel to our previously published data, where the prolate-ellipsoid method based on composition of three linear dimensions provided modestly better correlation with LAV obtained by electroanatomical mapping (i.e. CARTO) and, consequently, better prediction of LA enlargement than LAV based on planimetry [13]. However, an ellipsoid model demonstrated still low accuracy (large underestimation) of LAV_CT_. This finding stays in agreement with previous studies systematically identifying smaller LAV obtained by the prolate-ellipsoid method compared with 3D-ECHO or CARTO [6, 13, 25, 26].

High level of concordance between CT-assessed LA anatomy and LA CARTO maps has been already identified [15, 16]. LA shape assessment by electroanatomic mapping has also been shown to have acceptable correspondence to LAV assessed by intra-procedural 3D cone-beam CT angiogram [17]. In contrast to our data, all previously published studies have evaluated the relationships between linear dimensions [15] or surface-to-point distance [16, 17], instead to direct comparison of measured atrial volumes. Since LAV has been introduced as preferred parameter in LA size estimation, reevaluation of accuracy and precision of different methods of LAV assessment had become to be more relevant. Even though our study had found strong correlation (*r* = 0.8) between CARTO and the CT-derived LAV, the error reached relative range ~ 30%. The existence of wide range of agreement between LAV derived by CARTO and CT is novel finding. Moreover, in patients without SR during CT scan and electroanatomical mapping, overestimation of LAV_CT_ is more likely present. On the other hand, 3D electroanatomical reconstruction was insensitive to spherical deformation, when compared to biplane disc echocardiographic method.

In purposed study, dense, point-by-point electroanatomic LA reconstructions were built. All electroanatomical LA maps were created by experienced physicians. In case of high-density mapping, isolated inaccuracies in location of single points are usually mutually nullified, so high level of reproducibility would be presented. However, the volume of CARTO map is still dependent on how considerable segment of pulmonary vein antra, left atrial appendage, atrial region closed to mitral annulus is included to 3D reconstruction and how much stretch is applied on mobile interatrial septum leading to increase atrial volume. We speculate, that in case of advanced type of AF, especially when arrhythmia is ongoing, the 3D electroanatomical map is created more precisely in intention to identify more proarrhythmogenic substrate. This could be the most likely explanation for overestimation of LAV_CT_ by LAV_CARTO_ in case of absence of SR. This is in discrepancy with one previous small study showing that the heart rhythm has not influenced accuracy of fusion between CT and 3D electroanatomical map [16].

More indices describing LA shape remodeling have been introduced as predictors of effectiveness of catheter ablation [19, 27] or arrhythmia recurrence after DC cardioversion [28]. Site to IS, the asymmetry index describing ratio between volume of anterior portion of LA and total LAV has been proved as better prognostic tool [27]. For purpose of this technical study IS was chosen as marker of atrial remodeling as more simple method with high level of reproducibility.

The results of purposed study were partially presented as conference abstract at Euro Heart Care 2017 [29].

### Study limitations

The study has several limitations. First, it was retrospectively designed. The data collection was not independently monitored. Second, the study was single center study. Third, the time lag between imaging procedure and electroanatomical mapping was from one to three days in our study. However, it has been shown, that time lag less than 4 days from CT to electroanatomical mapping was the most likely not the factor responsible for integration error [17]. Only 3 patients manifested different rhythms among modalities. Fourth, LAV was acquired in different phase among the modalities. Both CT and CARTO obtained data in end-diastole. In contrast, ECHO used ventricular end-systole. Since diameters and planimetry are measured just before opening of mitral valve, atrial size should be maximal at this phase. We therefore speculate that underestimation of LAV by ECHO is not given by differences of time span of data acquisition. If the same phase of the heart cycle was used, underestimation of LAV with echocardiography would increase. If CT and CARTO were performed in systole their values would be larger than in diastole and hence bias would increase compared to echocardiography.

Finally, the results are valid in patients with non-valvular AF and cannot be probably converted to general population as well as to patients with valvular AF (severe mitral regurgitation or stenosis, presence of mitral valve prosthesis).

## Conclusions

Even though LA volumes evaluated by electroanatomical mapping have quite good accuracy, the precision is low. For volumes estimated by echocardiography, both precision and accuracy are low. The electroanatomical mapping is less sensitive to inaccuracy of LAV estimation predominantly driven by the magnitude of LA spherical remodeling. Referred bias between CT and CARTO-derived volumes is related to existence of sinus rhythm.

## Abbreviations

A4C: Apical four chamber view
AUC: Area under the curve
CI: Confidence interval
CT: Computed tomography
IQR: Interquartile range
IS: Index of sphericity
LA: Left atrium
LAD: Left atrial diameter in parasternal long-axis view (PLAX)
LAV: Left atrial volume
LAV_CARTO_: LAV obtained by electroanatomical mapping
LAV_CT_: LAV assessed by computed tomography
LAV_DISC_: LAV assessed by the biplane disc method
LAV_ELLIPSOID_: LAV assessed by the prolate-ellipsoid method
LAVi: LAV indexed to body surface area
LV EF: Left ventricular ejection fraction
SD: Standard deviation
SR: Sinus rhythm
